# Thoracic Surgeon Impressions of the Impact of the COVID-19 Pandemic on Lung Cancer Care—Lessons from the First Wave in Canada

**DOI:** 10.3390/curroncol28010092

**Published:** 2021-02-18

**Authors:** Roy A. Hilzenrat, Shaun A. Deen, John Yee, Kyle A. Grant, Ahmad S. Ashrafi, Shaun Coughlin, Anna L. McGuire

**Affiliations:** 1 Department of Surgery, Division of General Surgery, University of British Columbia, Vancouver, BC V5Z 1M9, Canada; roy.hilzenrat@gmail.com; 2 Department of Surgery, Division of Thoracic Surgery, University of British Columbia, Vancouver, BC V5Z 1M9, Canada; sdeen05@hotmail.com (S.A.D.); john.yee@vch.ca (J.Y.); kyle.grant@vch.ca (K.A.G.); logari1@yahoo.ca (A.S.A.); shaun.coughlin@gmail.com (S.C.); 3 Department of Surgery, Division of Thoracic Surgery, Interior Health Authority, Kelowna, BC V1Y 1T2, Canada; 4 Department of Surgery, Division of Thoracic Surgery, Vancouver Coastal Health Authority, Vancouver, BC V5Z 1M9, Canada; 5 Vancouver Coastal Health Research Institute, Vancouver, BC V5Z 1M9, Canada; 6 Department of Surgery, Division of Thoracic Surgery, Fraser Health Authority, Surrey, BC V5V 1Z2, Canada; 7 Department of Surgery, Division of Thoracic Surgery, Island Health Authority, Victoria, BC V8R 1J8, Canada

**Keywords:** lung cancer, thoracic malignancy, cancer care, COVID-19, Canada, survey

## Abstract

Background: COVID-19 has invariably changed the way lung cancer surgical care is provided in Canada. Despite relevant management guidelines, the way in which cancer care has been affected has yet to be described for thoracic surgical populations. Routine lung cancer physiologic and staging assessments are unique in that they are droplet producing and aerosolizing procedures. Our objective was to quantify the effect of the COVID-19 pandemic on surgical lung cancer care as perceived by practicing thoracic surgeons during the first wave of the pandemic in Canada. Methods: An electronic survey was distributed to members of the Canadian Association of Thoracic Surgeons. The survey was designed to determine surgeon perception of lung cancer preoperative care during the Canadian pandemic-instilled period of resource reallocation compared to standard care. Planned analyses were exploratory in nature; with count and frequency distributions of responses quantified. Results: Fifty-three thoracic surgeons completed the survey. Responses were collected from all Canadian provinces. Little change in access to preoperative imaging was noted. However, a significant decrease in access to lung function and bronchoscopy testing occurred. Pulmonary surgery was perceived to be lengthier with reduced operating theater availability. Despite decreased OR access, only 40% of surgeons were aware of respective institutional mitigation strategies. Summary: The COVID-19 pandemic has had an impact on standard lung cancer care preoperative workup. Further inquiry using institutional data is warranted to quantify its impact on cancer patient outcomes. Assessing the extent and effects of newly present barriers to standard lung cancer care is essential in forming appropriate mitigation strategies and planning for future pandemic waves.

## 1. Introduction

In 2019, the Canadian Cancer Statistics Advisory Committee estimated that 29,300 Canadians are diagnosed with lung cancer annually and 21,000 die from it [1]. Approximately 26% of all cancer-related deaths are attributable to lung cancer and this is more than colon, breast, and prostate cancer combined [1]. The only patients with a prospect of cure are those with early stage non-small cell lung cancer (NSCLC) who are amenable to surgical resection with curative intent [2]. In the midst of the novel coronavirus disease 2019 (COVID-19) pandemic, Canadian provinces had to make changes in health resource allocation in order to accommodate the first wave of COVID-19 patients in hospital, minimize virus transmission within hospital, and preserve human and personal protective equipment (PPE). It is estimated that cancer surgeries comprised 16% of outpatient surgeries and 5% of inpatient surgeries [3] delayed or canceled to accommodate the phased institutional preparations. In the era of COVID-19, routine lung cancer physiologic and staging assessments are unique in that they are droplet producing and aerosolizing procedures. These include pulmonary function testing (PFT), endobronchial ultrasound, and bronchoscopy [4].

The clinical team may further be exposed to aerosolized viral load intra-operatively, during double-lumen endotracheal tube placement and airway surgery, necessitating additional precautions [5]. The Society of Thoracic Surgeons, among several groups, have proposed recommendations for the care of surgical lung cancer patients during the COVID-19 pandemic [6,7]. The pre-operative workup and surgical pathway of lung cancer patients is inevitably affected; however, a crucial knowledge gap exists regarding precisely how this occurred in the Canadian public healthcare system, and how this may differ from published international recommendations [8]. Understanding the unique processes encountered by thoracic surgeons during the first wave of COVD-19 in Canada may allow mitigation of problems identified for subsequent waves.

We hypothesize that the coronavirus pandemic invariably changed the way lung cancer surgical care was provided in Canada. Despite the relevant recommendations on lung cancer patient management, the way in which cancer care has been affected has yet to be described for thoracic surgical populations. Our objective was to quantify the effect of the COVID-19 pandemic on surgical lung cancer care as perceived by practicing thoracic surgeons during the first wave of the pandemic in Canada.

## 2. Methods

Institutional research ethics board (IRB) approval was granted for this observational study (H20-01267), and participants provided informed consent. An English-language electronic survey was developed using the University of British Columbia (UBC) Qualtrics Survey Tool (Qualtrics, Provo, UT, USA), and distributed to members of the Canadian Association of Thoracic Surgeons (CATS). CATS is a national collaborative specialty network, with membership representative of Royal College of Canada Board certified Thoracic Surgeons across Canada. This includes approximately 90 surgeons in active thoracic clinical practice in Canada. The English-language survey consisted of 26 multiple-choice questions. Data were collected regarding thoracic surgeon demographics, the impact of the COVID-19 pandemic on lung cancer pre-operative physiologic and staging work-up, and access to the operating theatre for definitive treatment of early stage lung cancer. Over a 6-week period beginning 14 May 2020, the survey was distributed twice via the CATS electronic newsletter and twice via CATS listserv e-mail to active members. Planned analyses were descriptive and exploratory in nature while count and frequency distributions of survey responses were quantified.

## 3. Results

From the 90 eligible participants, a total of 53 (59%) survey responses were received. Two respondents declined consent to participate, and 3 provided partial responses. Characteristics of respondents are summarized in Table 1. Thoracic surgeons from all Canadian provinces provided responses, with the majority of replies from British Columbia (BC), Ontario, and Quebec. The majority of respondents practice in an academic clinical setting, and all participants treat lung cancer in their thoracic surgery practice. The study population is thus well versed in local institutional nuances regarding testing access to complete the standard of care physiologic and staging workup for lung cancer.

### 3.1. Impact on Pre-Operative Staging and Physiologic Work-Up

**Figure 1:**
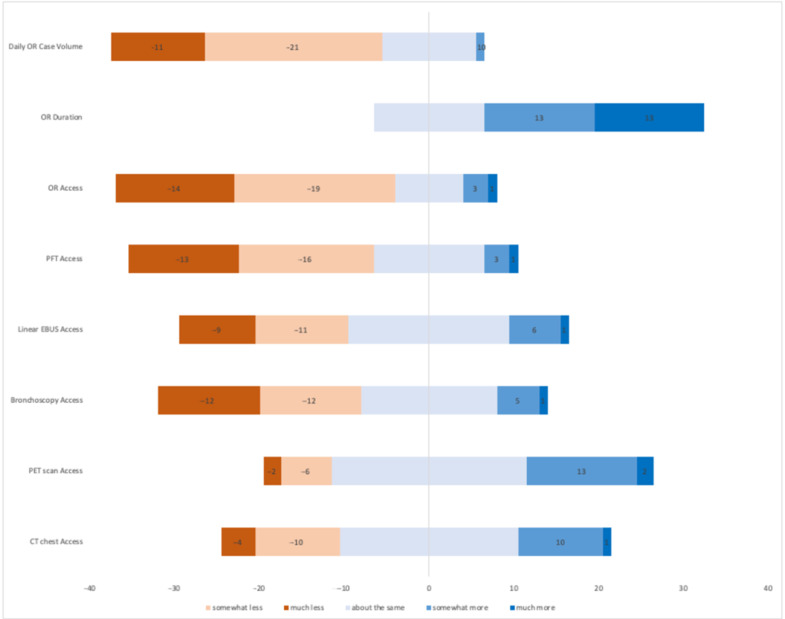
Diverging stacked bar chart displaying thoracic surgeon impressions regarding the impact of COVID-19 on lung cancer preoperative and operative variables. The *X*-axis displays respondent count, while the *Y*-axis lists the variable for thoracic surgeon consideration.

As displayed in Figure 1, access to pre-operative thoracic imaging remained similar compared to before the COVID19 pandemic. Thirty-two (70%) surgeons reported the same or increased access to computed tomography (CT) chest imaging at their institution, while 38 (82.6%) reported similar or increased access to flurodeoxyglucose positrion emission tomography(FDG-PET) nuclear medicine imaging. In contrast to thoracic imaging, surgeon access to aerosolizing procedures necessary to complete preoperative staging and physiologic assessment substantially decreased during the study period. As summarized in Figure 1, 40 (87%) and 39 (85%) of surgeons reported somewhat less or much less access to flexible bronchoscopy and linear endobronchial ultrasound respectively.

Thoracic Surgeons from across Canada communicated challenges in acquiring preoperative pulmonary function testing (spirometry and diffusion capacity). In the initial pandemic period, 29 (63%) reported much or somewhat less access to PFTs, while 13 (28.3%) reported no change in access. While exploring the reasons for change in access to PFTs, 9 surgeons reported the lab had temporarily closed, while 34 reported that bookings were limited to “essential” or “urgent” cases only.

### 3.2. Impact on Operating Theatre Access and Through-Put

The majority of thoracic surgeons communicated significant challenges with operating theatre access and lung cancer surgery daily throughput compared to the pre-pandemic period. A total of 33 (73%) respondents reported much and somewhat less access to the operating room for operative management of their lung cancer patients, while 8 (17.78%) noted no change. Operative times were notably increased as 32 (71%) respondents conveyed that standard pulmonary resection procedures required much or somewhat more time compared to the pre-COVID-19 era. Thirteen (29%) thoracic surgeons perceived no change in the average duration of time the patient was in the operating room from entry to exit.

The majority of surgeons attributed the increased operative time and decreased daily case throughput to new anesthesia COVID-19 safety protocols surrounding intubation (32/71%) or novel operative room air filtration protocols between cancer patients (28/62%).

Thirty-eight (84%) respondents noted that certain lung cancer patients were given priority when triaging OR access for pulmonary resection for numerous reasons. Twenty-seven (59%) surgeons either did not have or did not know whether their institution had a mitigation strategy to complete all delayed elective lung cancer surgeries. Nineteen (41%) surgeons knew of their hospital’s mitigation strategy, most of which constituted of increasing dedicated operating room access. 

## 4. Discussion

Resource reallocation and essential public health recommendations in light of the COVID-19 pandemic response have undeniably affected standard of care service delivery for thoracic malignancy patients. Despite little change in availability of pre-operative imaging, access to PFTs and invasive staging has greatly diminished as did available operating time. Lung cancer care necessitates a multi-disciplinary approach requiring close attention and effort to maintain access to standard of care preoperative assessment tools, all of which has been challenged by the COVID-19 pandemic.

### 4.1. Pre-Operative Lung Cancer Assessment in Face of COVID-19

Survey results indicate that institutional limitations during the first wave of the COVID-19 pandemic had little to no effect on pre-operative patient access to CT and PET scans. This may be due to the emphasis on ruling out COVID-19 infection in patients with respiratory symptoms and those at increased risk of COVID-19 complications such as the lung cancer population. An expert panel of pulmonologists, radiologists, and thoracic surgeons from the United States developed an approach to lung cancer imaging while health systems combat the increased COVID-19 associated care load [9]. At least 96% of the expert panel agreed to delay certain screening and surveillance imaging in the best interest of public health, patient safety, and cancer care. However, such recommendations should be taken into consideration with a focus on evidence-based patient care and an individualized balance of harm and benefit.

In contrast to imaging, thoracic surgeons across Canada noted a significant decrease in access to aerosolizing procedures such as PFTs; a critical test in lung resectability assessment. Absence of physiologic testing derails the course of standard cancer care provision, yet certain non-aerosolizing tests have been demonstrated to assess respiratory function and subsequent resectability. The six-minute walk test (6MWT) is a simple evaluation of exercise performance primarily used in evaluating risk of hospitalization in pulmonary disease. However, it has also been demonstrated to predict postoperative pulmonary complications [10]. Similarly, inability to complete an incremental shuttle walk test distance greater than 400 m has been associated with increased risk of perioperative mortality and cardiopulmonary complications in the major thoracic surgery population [11]. The stair climb test is yet another popular low-tech assessment of pulmonary function. A prospective series of 640 lobectomy and pneumonectomy patients had demonstrated a twofold increase in cardiopulmonary complications and 13-fold increase in mortality for patients unable to climb stairs to the altitude equivalent of 22 m or more [12]. No groups have yet described their approach in mitigating the challenge of decreased access to PFTs, and may consider the described low-tech tests as possible alternatives in select cases.

### 4.2. Operative Lung Cancer Care

With decreased access to preoperative assessment tools and elective operating room (OR) time, challenges in meeting the standard of lung cancer care were inevitable. Despite the increased hospital burden of COVID-19, we maintain that the standard of care for lung cancer patients must remain unchanged. Certain expert groups have listed recommendations to maintain high quality care during this time of decreased resources and patient-physician interaction. Preliminary recommendations by thoracic surgeons from China focus on clinical and pathology findings suggestive of necessitating emergent surgery [13]. Patients with resectable central pulmonary lesions accompanied by massive hemoptysis, and patients with major airway involvement accompanied by severe dyspnea, should clearly undergo resection emergently. They note that aerosolizing procedures, such as simple bronchoscopy and washes without EBUS-TBNA, are not recommended during the outbreak phase. An expert panel from Switzerland have similarly developed an algorithm to allocate lung cancer patients into categories of surgical urgency [7]. An integrated risk classification is based on patient risk of cancer progression (based on tumor TNM staging) and of COVID-19 infection. Patients with high risk of progression and low risk of COVID infection are recommended to undergo definitive oncological treatment. In contrast, those with high risk of COVID infection must have a discussion with their cancer care team regarding timing of treatment in relation to pandemic severity.

The American College of Surgeons, in collaboration with the Society of Thoracic Surgeons and American Association for Thoracic Surgery, recommends deferring treatment of lung cancer <2 cm (T1a, T1b). Adenocarcinoma and squamous cell carcinoma of the lung have the potential of advancing from early stage IA disease to advanced IIIB disease within 1.17 and 2.5 years, respectively [14]. A lengthy delay in follow up imaging or treatment may have significant implications on the curability of early stage lung neoplasms.

The Quebec Lung Cancer Network has also published its recommendations in treating lung cancer during the COVID-19 pandemic [15]. Their consensus statement highlights that select treatments should be prioritized, such as those for stages I-III NSCLC and malignant or symptomatic mediastinal tumors. Taking into account the availability of certain treatments, alternatives should be considered such as stereotactic radiation therapy and hypofractionated radiotherapy. These recommendations generally align with our survey results regarding perceived patient prioritization in Canadian hospitals. The most common criteria for patient prioritization as per the survey are large tumor diameter, neoadjuvant therapy previously conducted, suspected or confirmed N1 disease, central tumor location, and more advanced American Joint Commission on Cancer (AJCC) stage. Close monitoring of risk-benefit must be regularly conducted in order to continually reassess such recommendations as the pandemic progresses.

Subgroups of lung cancer patients have been treated either surgically or with antitumor treatment prior to the pandemic and are in their follow-up stage of cancer care. A group of Brazilian thoracic oncologists have organized recommendations for the management of patients in follow-up based on expert opinion given the scarcity of reliable data related to this topic [16]. In brief, they recommend postponing follow-up imaging by variable lengths of time, from 6 months to 1 year, depending on initial cancer stage, cancer type, intent of treatment, time of treatment, and patient symptoms. Cancer centers should organize similar expert groups to elect the acceptable postponement time of follow-up testing in their respective region and population, if necessary. Current recommendations fail to account for the lengthy presence of COVID-19 and its implication on health systems and on cancer patients. Therefore, close monitoring of risk-benefit must be regularly conducted in order to continually reassess such recommendations as the pandemic progresses.

### 4.3. Mitigation Strategies

The Canadian Institute for Health Information estimates 100,000 inpatient and 375,000 day surgeries were planned from February to May 2020 [3]. Considering that cancer surgeries constitute 5% of all Canadian day surgeries and up to 19% of inpatient surgeries [3], tens of thousands of cancer surgeries are at risk of cancellation in the face of COVID-19 driven resource reallocation. Between March 16 and May 18, the British Columbia Ministry of Health declared that over 30,298 elective surgeries have been delayed or canceled due to the COVID-19 pandemic [17]. The Financial Accountability Office of Ontario reported 52,700 canceled hospital procedures from 15 March to 22 April 2020 with an additional 12,200 procedures being delayed each week Ontario hospitals continue to postpone elective surgery [18].

Lung cancer patients undoubtedly make up a proportion of these elective surgery cancellations, creating a backlog of patients requiring curative resection. Our study revealed a substantial decrease in OR access and increased OR times. Only 40% of surveyed thoracic surgeons confirmed the development of an institutional mitigation strategy to withstand increased operative waitlists. Provinces have traditionally governed wait lists on the basis of equitable access depending on a patient’s condition using a multi-staged triage system. Provincial governments and hospitals will be required to re-evaluate their triage systems and form a mitigation strategy to accommodate the expected encumbrance of canceled surgeries. To date, no province has issued a recovery plan. Lung cancer care is a multidisciplinary effort which also includes diagnostic imaging, clinical laboratories, and post-anesthesia care units. All aspects of hospital capacity and cancer care must be considered to organize an achievable and effective mitigation strategy.

### 4.4. Is a Path to Normalcy in Pre-Operative Assessment Possible?

The COVID-19 pandemic has led to the collapse of economies, livelihoods, systems of education, societal structure, and what we refer to as normal quotidian life. The Lancet Microbe and public health experts such as the director-general of the World Health Organization have stated that there will be no return to the “old normal” in the near future, if at all [19]. A conglomerate of Canadian experts in tropical medicine, infectious disease, and global health have recently put forth three possible scenarios once a vaccine is distributed for COVID-19 [20]. Scenario one is the rollout of a vaccine that prevents nearly all further human spread (the best-case scenario, similarly to the vaccine of hepatitis A, measles, and polio). The two remaining scenarios are such that either a vaccine prevents some degree of human spread or that it only decreases severe illness and death, without affecting spread. Although the scenario of a vaccine partially reducing spread being the most likely, it would be unwise to assume this as a certain future. Due to the indeterminable length of COVID-19 burden, for the first time for many Canadians, the future is uncertain. Similarly is true for Canadian physicians and the environment in which they care for their patients. As aforementioned, several steps and considerations must be taken to ensure persistent, high quality cancer care in the context of COVID-19. However, we must equally begin evaluating what the future of lung cancer care may look like.

Firstly, we must be aware that COVID-19 disproportionately impacts lung cancer patients. A multitude of studies reveal that lung cancer patients are at increased risk of contracting COVID-19, with associated increased mortality compared to the general population [21,22,23]. Thus, future lung cancer care strategies should be particularly protective of patients either at increased risk of infection or in high risk regions. Older age seems to negatively impact mortality as does the time from last antitumor treatment [21,24], suggesting that patients with such risk factors should particularly maintain social distancing and avoid non-emergent hospital visits, as directed by their cancer management team. Despite the pandemic, improvement in lung cancer mortality has been reported in 2020 and is believed to be on account of major developments in the treatment of advanced-stage NSCLC with tyrosine kinase inhibitors and immune checkpoint inhibitors [25]. Although rigorous measures are needed to protect lung cancer patients from COVID-19, barriers to timely cancer care may lead to increased cancer-related mortality. Therefore, it is pivotal to develop a “new normal” for lung cancer care, including individualized treatment regimens depending on infection and associated mortality risk. 

The global consortium, TERAVOLT (The Thoracic Cancers International COVID-19 Collaboration) has begun studying which lung cancer patients may be at increased risk of COVID-19-related mortality with early results showing no added risk with immunotherapy [24]. Similar studies that will facilitate patient risk stratification based on cancer stage, therapy choice, and comorbidities are crucial in appropriately protecting thoracic cancer patients from COVID-19. The path back to normalcy should also include new evaluations as to the benefit of preventative strategies, particularly smoking in lung cancer patients. TERAVOLT has shown in multivariable analysis that smoking habits are the only significant associated factor with mortality in the thoracic cancer population.

### 4.5. Limitations

Our report is not without limitations. These include underrepresentation from thoracic surgeons in community practice. Similarly, there is overrepresentation of surgeons from Quebec, Ontario, and British Columbia, which collectively make up 70% of survey responses. Although these three provinces contain the greatest number of employed thoracic surgeons as per the 2019 Canadian Medical Association thoracic surgery specialty profile, our study aims to portray the Canada-wide experience. Surgeons within the same milieu may answer differently to questions regarding relative change, such as those included in the survey (e.g., “somewhat less”), leading to a degree of response variability in the context of subjective experiences. Despite the acceptable response rate to our survey, a non-response bias cannot be disregarded given the voluntary nature of our survey. We further acknowledge that our report represents a cross-sectional description during a highly dynamic pandemic response, which along with selection bias, may affect the external validity of our results. Lastly, this report indicates the necessity for institutional data collection and review in order to metric subjective physician perceptions against administrative routine data sources. The accumulation of such national data during the first wave of the COVID-19 pandemic response with the Canadian Institute for Health Information (CIHI) is crucial in this regard.

## 5. Conclusions

Our report characterizes the challenges faced by Canadian thoracic surgeons and lung cancer patients during the first wave of the COVID-19 pandemic. Comprehensive care involves not only access to the operating theatre for definitive lung cancer treatment, but also access to tests of physiologic assessment and clinical staging in order to determine resectability and operability.

The concern is that suboptimal access to all aspects of the preoperative care pathway will result in issues with meeting the standard of care. Alternative tests exist with inadequate validation when compared to gold standards. Although several expert groups have published recommendations on management of lung cancer during the COVID-19 pandemic, they are based primarily on expert opinion with questionable strength of evidence. Ongoing study is crucial to further quantify the impact of pandemic response preparations for lung cancer patients, and to determine optimal clinical mitigation strategies that maintain standard of oncologic care for future COVID-19 waves in Canada.

## Figures and Tables

**Table 1:** Respondent characteristics.

Characteristic	*N* (%)
Age (years)	
25–34	2 (4.35)
35–44	20 (43.48)
45–54	17 (36.96)
55–64	6 (13.04)
≥65	1 (2.17)
Sex	
Male	32 (69.57)
Female	12 (26.09)
Prefer not to say	2 (4.35)
Practice Setting	
Academic Hospital	40 (87)
Community Hospital	6 (13)
Years in Thoracic Surgical Practice	
≤5	22 (43.14)
6–10	5 (9.8)
11–15	6 (11.76)
16–20	10 (19.61)
21–25	6 (11.76)
>25	2 (3.92)
Annual pulmonary resections for lung cancer	
≤50	6 (13.04)
51–100	24 (52.17)
101–150	7 (15.22)
151–200	5 (10.87)
>200	4 (8.70)
Province of Thoracic Surgical Practice	
British Columbia	14 (27.45)
Alberta	3 (5.88)
Saskatchewan	3 (5.88)
Manitoba	3 (5.88)
Ontario	12 (23.53)
Quebec	10 (19.61)
Maritime Province *	6 (11.76)

* Newfoundland and Labrador, New Brunswick, Nova Scotia, Prince Edward Island.
